# Multi-Layered, Corona Charged Melt Blown Nonwovens as High Performance PM_0.3_ Air Filters

**DOI:** 10.3390/polym13040485

**Published:** 2021-02-04

**Authors:** Xing Zhang, Jinxin Liu, Haifeng Zhang, Jue Hou, Yuxiao Wang, Chao Deng, Chen Huang, Xiangyu Jin

**Affiliations:** 1 Engineering Research Center of Technical Textiles, Ministry of Education, College of Textiles, Donghua University, Shanghai 201620, China; tulip_90@163.com (X.Z.); drliujx@163.com (J.L.); 1159117@mail.dhu.edu.cn (J.H.); baoer52121@163.com (Y.W.); hc@dhu.edu.cn (C.H.); 2 College of Textile and Clothing, Nantong University, Nantong 226019, China; zhanghf@ntu.edu.cn; 3 Joint International Research Lab of Lignocellulosic Functional Materials and Provincial Key Lab of Pulp and Paper Sci & Tech, Nanjing Forestry University, Nanjing 210037, China; cdeng@njfu.edu.cn

**Keywords:** multi-layer, melt blown, corona charging, air filter

## Abstract

Particulate matter (PM) and airborne viruses bring adverse influence on human health. As the most feasible way to prevent inhalation of these pollutants, face masks with excellent filtration efficiency and low press drop are in urgent demand. In this study, we report a novel methodology for producing high performance air filter by combining melt blown technique with corona charging treatment. Changing the crystal structure of polypropylene by adding magnesium stearate can avoid charge escape and ensure the stability of filtration performances. Particularly, the influence of fiber diameter, pore size, porosity, and charge storage on the filtration performances of the filter are thoroughly investigated. The filtration performances of the materials, including the loading test performance are also studied. The melt blown materials formed by four layers presented a significant filtration efficiency of 97.96%, a low pressure drop of 84.28 Pa, and a high quality factor (QF) of 0.046 Pa^−1^ for paraffin oil aerosol particles. Meanwhile, a robust filtration efficiency of 99.03%, a low pressure drop of 82.32 Pa, and an excellent QF of 0.056 Pa^−1^ for NaCl aerosol particles could be easily achieved. The multi-layered melt blown filtration material developed here would be potentially applied in the field of protective masks.

## 1. Introduction

Particulate matter (PM) and airborne viruses have gained increasing attention in air filtration field [1]. Many studies have proved that these pollutants are pose serious threats to human health [2]. As a result of this, more and more people have realized the importance of wearing masks, especially during the haze days and the prevalence of respiratory infectious diseases [3]. As the most feasible way to prevent inhalation of these pollutants, face masks with excellent filtration performances and high comfort are in urgent demand.

Masks are usually composed of nonwovens produced via spunbond, needle-punch, through-air bonding, electrospinning, and melt blown technologies [4,5,6]. Among them, spunbond, needle-punch and through-air bonding nonwovens usually serve as the cover layer or the inner lining of the mask. Since the fiber diameter of these nonwovens are tens of micrometers, they are only suitable for coarse filtration [7,8,9]. Electrospun membranes have been studied intensively in recent years, for that the small diameter of electrospun fibers are conducive for a high filtration efficiency. However, there are still some disadvantages to restrict the development of electrospun material, such as high pressure drop, low strength, solvent residue, and difficulties for industrialized manufacturing [10,11,12]. For these reasons, till now, melt blown nonwovens are the most successful materials for constructing the core filter of the mask and the quality of masks is mostly determined by the filtering performance of melt blown nonwovens.

In general, melt blown nonwoven has a three-dimensional (3D) network structure, small pore size but large porosity, enabling ideal filtration performances. Furthermore, when additional electret treatments are involved, electrical charges can be readily introduced onto the melt blown nonwovens, so that the strong electric absorption from the electric charges provides melt blown nonwovens with the capacity to efficiently capture fine particles [13,14,15]. For instance, Hassan et al. fabricated electret melt blown materials with a filtration efficiency of 99.978% and a pressure drop of 280 Pa [16]. Albert et al. prepared polypropylene (PP) melt blown nonwovens with a filtration efficiency of 99.253% and the pressure drop was 173 Pa [17]. While these works all led to high filtration efficiency, the pressure drops are relatively high. Since high breathing resistance would make the wearers (children and elder people in particular) feel uncomfortable, or even causing breathing difficulties, the development of melt blown nonwovens with low pressure drop is of important research value. The most penetrating particle size (MPPS) of a traditional filter is about 0.3 μm. Due to its smaller particle size, PM_0.3_ is stronger than PM_2.5_ in terms of penetration, air residence time, and diffusion width. PM_0.3_ can not only reach deep into the lungs, but also enter the olfactory area of the brain through the nasal cavity, which is far more harmful to health than PM_2.5_ [18]. During the filtration process, the inertial impaction mechanism is prevalent for PM_2.5_, but for PM_0.3_, Brownian diffusion, gravity, inertial impaction, and interception mechanism are not significant [19]. The electrostatic force plays a more significant role to capture PM_0.3_ [20]. Our previous studies have found that the existence of magnesium stearate (MgSt) could effectively improve the crystallinity and the electrostatic force of melt blown materials [21]. This is because MgSt could serve as a nucleating agent to avoid charge escape and ensure the stability of filtration performances. The crystal structure of melt blown nonwovens containing MgSt showed smaller average crystallite size and more crystal granules, and thus increasing the crystal interface of PP fibers. Since a large number of charges can be stored in the interface between crystalline and amorphous regions, the increase of crystal interface is beneficial for the storage of electrical charges [8]. However, previous reports lack the in-depth investigation of material structure and filtration for paraffin oil aerosol particles, while the above properties have significant impacts on the actual use of melt blown nonwovens.

Herein, we report a novel methodology for producing a high performance air filter by combining melt blown technique with corona charging treatment. The multi-layered nonwoven materials exhibit low pressure drop and high efficiency for PM_0.3_. Particularly, the influence of fiber diameter, pore size, porosity, and charge storage on the filtration performances of the filter are thoroughly investigated. The filtration performances of the materials, including the loading test performance are also studied.

## 2. Materials and Methods

### 2.1. Materials

PP pellets with a melt flow index (MFI) of 36 g 10 min^−1^ were provided by Shanghai SECCO Petrochemical Company Limited (Shanghai, China). The conditions of MFI test were in accordance with ISO 1133. The temperature was 230 °C and the load was 2.16 kg. MgSt powders with an MFI of 400 g 10 min^−1^ were purchased from Sinopharm Chemical Reagen Company Limited (Shanghai, China).

Then, 199 g of PP and 1 g of MgSt were fed into the hopper from different screws at the same time. The feeding speeds of PP and MgSt were controlled as 30 r/min and 2 r/min, respectively. All materials were mixed together for 10 min and then poured fast into the screw extruder.

### 2.2. Fabrication of Melt Blown Nonwovens

**Figure 1:**
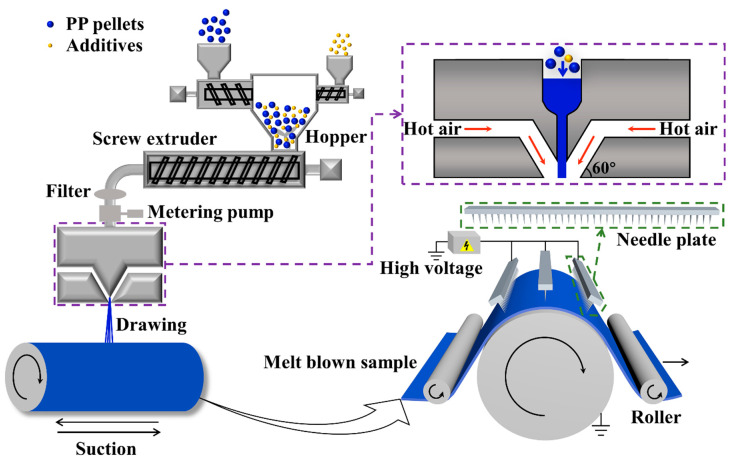
Fabrication process of the melt blown nonwovens and corona charging treatment.

A melt blown pilot line with 142 spinnerets (diameter of each spinnerets = 0.25 mm) at the Nonwoven Research and Development Center of Donghua University (Shanghai, China) was used to produce the PP melt blown nonwovens with areal density of 10 g m^−2^. The schematic illustration of the fabrication process of the melt blown nonwovens is exhibited in Figure 1. The air drawing angle was 60°.

The screw speed, screw temperature, air temperature, air pressure, and die-collector distance are the main processing parameters. For PP pellets with low melt index, setting higher processing temperature that is lower than its thermal degradation temperature, could effectively enhance the melt fluidity, which is conducive to acquiring finer fibers. Herein, thermal degradation temperature of PP pellets has been tested via TGA and DTG, indicating the processing temperature (280 °C–345 °C) is reasonable. Therefore, in this paper, the temperature of screw zones 1, 2 and 3 were 280 °C, 340 °C, and 345 °C, respectively. The parameters will obviously influence the performances of productions, and they are listed in Table 1.

### 2.3. Corona Charging

The corona charging was conducted by a dual-charging apparatus as presented in Figure 1. The apparatus is composed of a high voltage power supplier, and three arrays of electrode needles. After applying a high voltage between the needles and the grounded plate electrode, ions were generated and deposited on the melt blown samples. The process was carried out at ambient temperature of 25 ± 2 °C and a relative humidity of 40 ± 5%. The applied voltage and the charging time were 80 kV and 10–40 s, respectively. The charging distance between the needles and the grounded plate electrode was set to 100 mm.

### 2.4. Characteristics of the Manufactured Nonwovens

The surface morphology of melt blown nonwovens was investigated by scanning electron microscope (SEM, TM 3000, Hitachi Ltd., Tokyo, Japan) after sputter-coating the samples with gold. Fiber diameter and its distribution were determined by randomly measuring 100 fibers from SEM images, using the software Nano Measurer 1.2.5.

The pore size and distribution of melt blown nonwovens were measured by a capillary flow porometer (CFP-1100AI, Porous Materials Inc., Ithaca, NY, USA) through the bubble point method.

The porosity was calculated as follows:

(1) $$P=\left(1-\frac{m}{\rho \;\cdot \;\delta }\right)$$

where *m*, *ρ*, and *δ* represent the areal density, fiber density, and nonwoven thickness, respectively.

The surface potential of melt blown nonwovens was measured by a non-contacting electrostatic voltmeter (TREK-542A-2-CE, TREK Inc., Lockport, NY, USA).

### 2.5. Filtration Performances of Manufactured Nonwovens

The filtration performance of melt blown nonwovens was measured by an automated filter machine (TSI 8130, TSI Instruments Co. Ltd., Shoreview, MN, USA). The mass median diameter of sodium chloride aerosol particles was 0.26 μm, and the geometric standard deviation of the particles was less than 1.83. The mass median diameter of paraffin oil aerosol particles was 0.33 μm, and the geometric standard deviation of the particles was less than 1.6. Aerosol particles passed through melt blown nonwovens with an effective area of 100 cm^2^ at the air flow rate of 85 L min^−1^. The filtration efficiency was calculated by measuring the concentration of aerosol particles in the upstream and downstream of melt blown nonwovens. Filtration efficiency was calculated as follows:

(2) $$\eta =\frac{\varepsilon_{1}-\varepsilon_{2}}{\varepsilon_{1}}\times 100\%$$

where *η*, *ε_1_*, and *ε_2_* represent filtration efficiency, concentration of aerosol particles in the upstream and downstream of melt blown nonwovens, respectively. The pressure drop of melt blown nonwovens was measured by a flow gauge and two electronic pressure transmitters. Loading filtration performances of melt blown nonwovens were continuously tested under the air flow rate of 85 L min^−1^.

A trade-off parameter of quality factor (QF) between filtration efficiency and pressure drop was adopted to comprehensively evaluate the filtration performance of a given filtration medium. QF of filtration medium is defined by the following formula:

(3) $$QF=\frac{\ln\frac{1}{1-\eta }}{\Delta p}$$

where Δ*p* represents the pressure drop.

## 3. Results

### 3.1. Design and Fabrication of Melt Blown Nonwovens

The target of this study is to fabricate a filtration material with high filtration efficiency and ultralow pressure drop. To this end, we designed the filtration material based on the following three criteria: (1) the fiber of the filtration material must be fine, and the pore size must be small to achieve high filtration efficiency; (2) the porosity of the filtration material must be large enough to ensure an ultralow pressure drop; (3) the filtration material must have a 3D fluffy structure to accommodate dusts. These three objectives could be satisfied by melt blown technology, in which polymer melts are stretched by high-speed hot air to form 3D nonwovens with fine fibers, small pore size, and high porosity.

**Figure 2:**
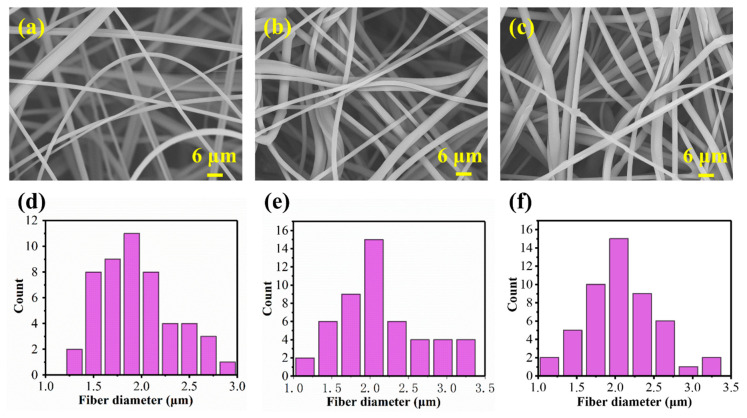
Morphology and diameter of melt blown nonwovens with different collecting time. SEM images of the surface of (**a**) MB-A*1, (**b**) MB-B*1, and (**c**) MB-C*1. Fiber diameter distribution of (**d**) MB-A*1, (**e**) MB-B*1, and (**f**) MB-C*1.

By regulating collecting time, three types of the melt blown nonwovens were fabricated under the same processing parameters. These nonwovens were named as MB-A*1, MB-B*1, and MB-C*1, and the areal densities were 10, 20, and 40 g m^−2^, respectively. As shown by the representative SEM images in Figure 2a–c, the fibers in all samples were randomly distributed, and there is no significant difference in the average diameters (Figure 2d–f). The fiber diameters of MB-A*1, MB-B*1, and MB-C*1 were 1.91 ± 0.22, 1.94 ± 0.23, and 1.96 ± 0.41 μm, respectively. These results are as expected, because parameters affecting fiber diameter, including screw speed, air pressure, and die-collector distance were not changed throughout the melt blown process.

**Figure 3:**
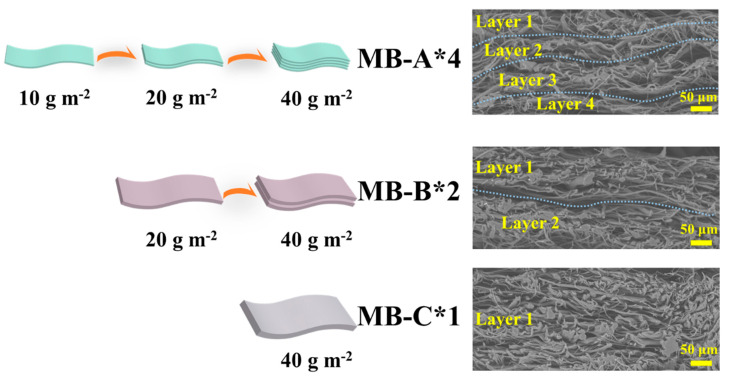
Structure diagram and SEM images of the cross section of MB-A*4, MB-B*2, and MB-C*1.

In order to improve filtration performance, a self-developed corona charging technology was applied. The samples were charged at a voltage of 80 kV and the charging distance between electrodes was 100 mm. The time used for electret treatment of sample MB-A*1, MB-B*1, and MB-C*1 was 10, 20, 40 s, respectively. During the charging process, samples can assemble together by the electrostatic force. As shown in Figure 3, melt blown nonwovens having the same density of 40 g m^−2^ but different numbers of fiber layers were acquired. MB-A*2, MB-A*4, and MB-B*2 represent the composited filter with two layers of MB-A*1 sample, four layers of MB-A*1 sample, and two layers of MB-B*1 sample, respectively.

**Figure 4:**
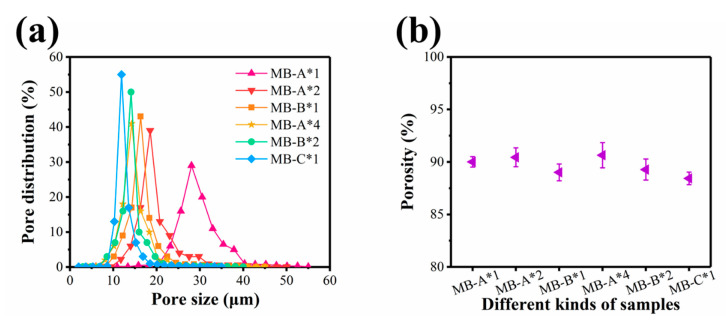
(**a**) Pore size and pore size distribution curves and (**b**) porosity.

The pore size, pore size distribution, and porosity of sample were further investigated. As shown in Figure 4a, pore sizes of MB-A*1, MB-A*2, MB-B*1, MB-A*4, MB-B*2, and MB-C*1 were in the range of 2–55 μm. Although the densities of MB-A*4, MB-B*2, and MB-C*1 are identical, samples containing more layers show larger pore size. It can be seen from Figure 4b that the porosities of MB-A*1, MB-A*2, MB-B*1, MB-A*4, MB-B*2, and MB-C*1 were 90.0%, 90.4%, 89.0%, 90.6%, 89.3%, 88.4%, respectively. The results showed that the porosity of six kinds of nonwovens was large, and there was no significant difference, which conformed to the characteristics of melt blown nonwovens with high porosity. Obviously, for samples of the same areal density, the increase of layer number leads to a more fluffy structure, making us speculate that the multi-layered melt blown nonwovens may possess better filtration performances.

**Figure 5:**
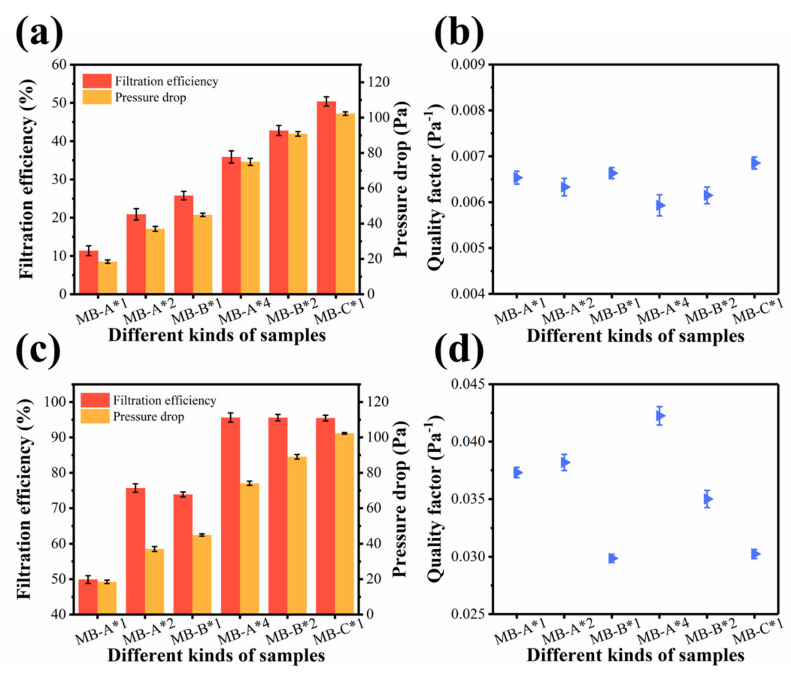
(**a**) Filtration performances and (**b**) quality factors of different kinds of samples before corona charging. (**c**) Filtration performances and (**d**) quality factors of different kinds of samples after corona charging.

To validate this assumption, filtration performances of the melt blown nonwovens were systematically investigated by using the charge neutralized sodium chloride (NaCl) particles. As shown in Figure 5a, before corona charging, the filtration efficiencies of the MB-A*1, MB-A*2, MB-B*1, MB-A*4, MB-B*2, and MB-C*1 were 11.42%, 20.91%, 25.79%, 35.94%, 42.83%, 50.37%, while the corresponding pressure drops were 18.52, 37.04, 44.98, 74.97, 90.85, 102.31 Pa, respectively.

As illustrated in Figure 5b, QFs of samples are basically at the same level. This is because NaCl particles were mainly captured by physical sieving and interception at this stage. Since the pore size and fiber diameter of the samples were basically the same, the difference in filtration performances was not obvious.

The charging process provides the nonwovens with extra electrostatic forces to capture fine particles. As shown in Figure 5c, after corona charging, the filtration efficiencies of samples increase significantly (49.92%, 75.73%, 73.91%, 95.63%, 95.58%, 95.47%) when compared with uncharged samples, while the pressure drop remains unchanged. Moreover, in the absence of dielectric breakdown, corona charging did not damage the structure of the material, and thus did not affect the pressure drop. The sharp increase of QF values (0.037, 0.038, 0.030, 0.042, 0.035, 0.030 Pa^−1^) confirms the improvement made by corona charging (Figure 5d). It also indicates that the four layered structure is optimal.

### 3.2. The Mechanism for the Improvement of Filtration Efficiency

**Figure 6:**
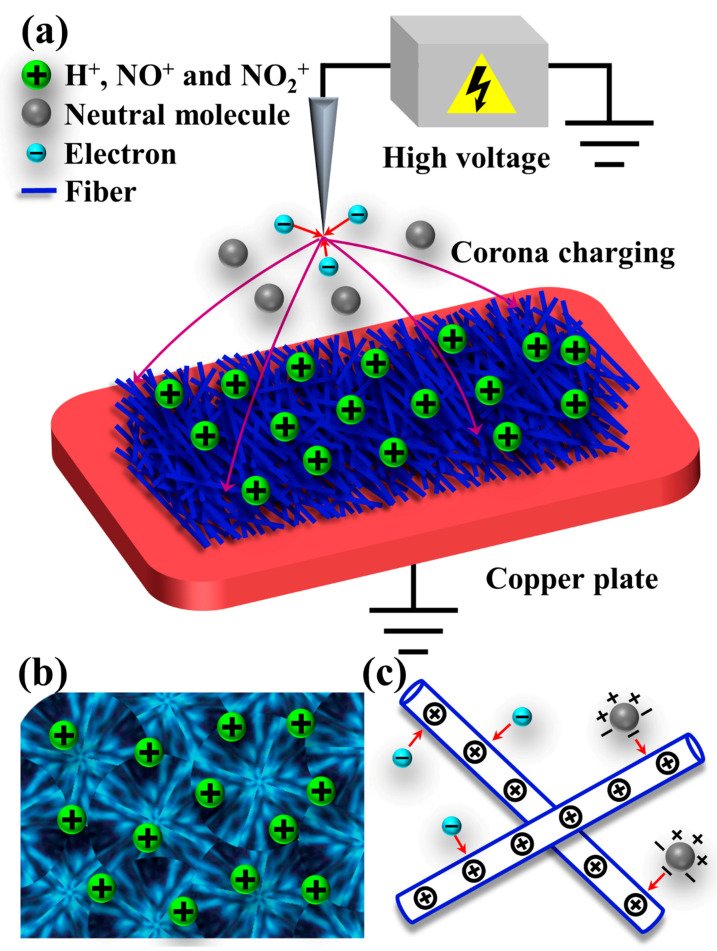
(**a**) The schematic diagram of corona charging process, and surface charges. Space charges stored in (**b**) defects of polymer. (**c**) Polarization charges generated by strong electric field.

Figure 6a illustrates the process of corona charging. When a high positive voltage was applied to the needle electrode, the electric field around the needle tip would be distorted. The distorted electric field caused neutral molecules in the air to be ionized as H^+^, NO^+^, and NO_2_^+^ ions [22]. Due to the large potential difference between the high voltage electrode and the ground electrode, these positive ions moved toward the ground electrode and deposited on the nonwovens to form surface charges [23]. In this process, structural defects and impurity defects of the polymer are also important, as they can provide extra storage locations for space charges (Figure 6b) [24]. In addition, as revealed in Figure 6c, when the air flow passed through the filter material, the Coulomb force of the electrostatic field not only effectively attracts the charged particles in the air flow, but also polarizes and eventually captures the neutral particles [25].

**Figure 7:**
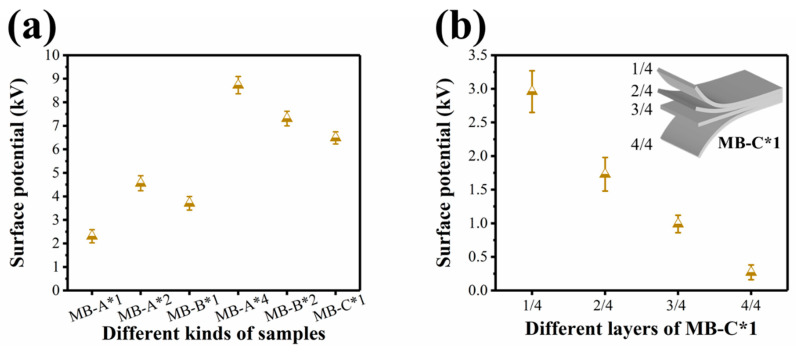
(**a**) Surface potential of different kinds of samples, and (**b**) different layers of sample MB-C*1.

To investigate the effect of charge distribution on filtration efficiency, we tested the surface potential of these melt blown nonwovens. As revealed in Figure 7a, the surface potentials of the MB-A*1, MB-A*2, MB-B*1, MB-A*4, MB-B*2, and MB-C*1 were 2.31, 4.56, 3.71, 8.73, 7.31, 6.49 kV, respectively. In addition, MB-C*1 was evenly divided into four layers and the four-layers structure of was inset in Figure 7b. The surface potentials of four layers of MB-C*1 were 2.94, 1.73, 0.99, 0.27 kV, respectively. This demonstrated that the filtration efficiency of MB-A*4 sample was higher than those of other samples, because that the former has a higher total charge amount, and charge was mainly stored on the surface or close to the surface of the nonwovens [26].

### 3.3. Removal of Paraffin Oil by Multi-Layered Melt Blown Nonwovens

**Figure 8:**
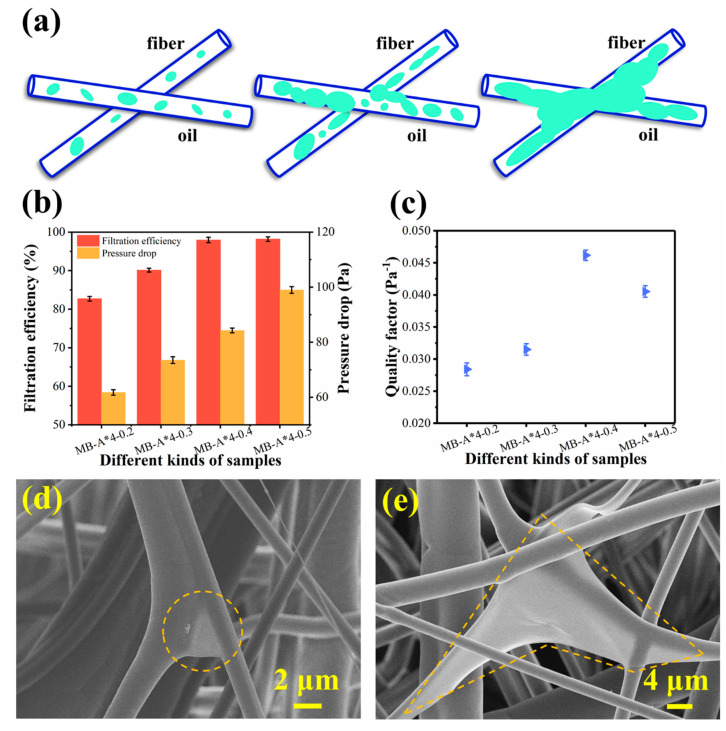
(**a**) The filtration process of melt blown materials for the paraffin oil aerosol particles. (**b**) Filtration performances and (**c**) quality factors of different kinds of samples after corona charging. (**d**,**e**) SEM images of melt blown materials after paraffin oil aerosol filtration test.

Figure 8a shows the filtration of paraffin oil aerosol particles with mass mean diameter of 0.33 mm by melt blown materials. Four kinds of samples were prepared by adjusting the air pressure. MB-A*4-0.2, MB-A*4-0.3, MB-A*4-0.4, and MB-A*4-0.5 represent that the air pressures were 0.2 MPa, 0.3 MPa, 0.4 MPa, and 0.5 MPa, respectively. The fiber diameters of MB-A*4-0.2, MB-A*4-0.3, MB-A*4-0.4, and MB-A*4-0.5 were 2.31, 1.94, 1.63, and 1.55 μm, respectively. Before corona charging, the filtration efficiencies of MB-A*4-0.2, MB-A*4-0.3, MB-A*4-0.4, and MB-A*4-0.5 were 24.69%, 28.58%, 34.23%, and 35.75%, respectively. As shown in Figure 8b, after corona charging, the filtration efficiencies of MB-A*4-0.2, MB-A*4-0.3, MB-A*4-0.4, and MB-A*4-0.5 were 82.73%, 90.13%, 97.96%, 98.19%, while the corresponding pressure drops were 61.74, 73.50, 84.28, 98.98 Pa, respectively. This suggests that electret treatment can significantly improve the filtration performance of melt blown nonwovens. The QF values of materials had significant difference, and the sample of MB-A*4-0.4 shows the highest value of 0.046 Pa^−1^ (Figure 8c). It indicates that the four layered structure under 0.4 MPa is optimal. It can be also observed from Figure 8d and e that paraffin oil was intercepted on the surface and the cross of the fibers.

### 3.4. Evaluation of Air Filtration Performances

**Figure 9:**
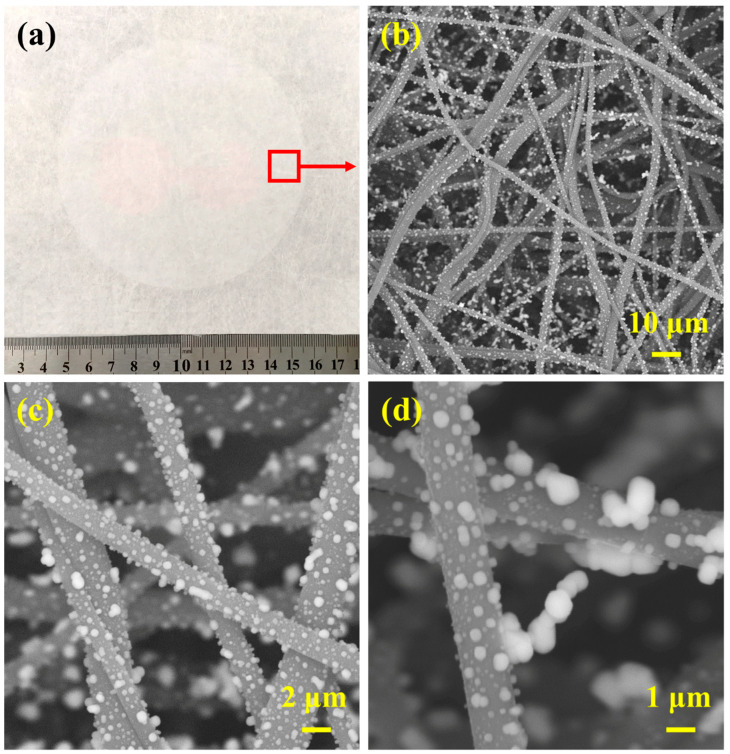
(**a**) Photograph and (**b**–**d**) SEM images of melt blown materials after filtration loading test.

In addition to the sufficient filtration of paraffin oil aerosol particles, our nonwoven filters also show high removal rate against NaCl aerosol particles with mass mean diameter of 0.26 mm. It can be clearly observed from Figure 9 that considerable NaCl particles were successfully intercepted on both the surface and the interior of the nonwovens (Figure 9b–d).

**Figure 10:**
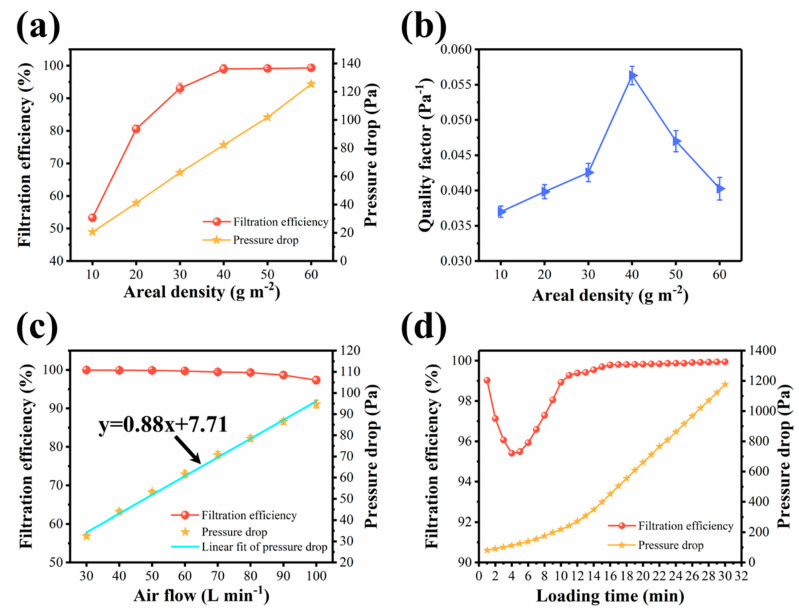
(**a**) Filtration efficiency and pressure drop, and (**b**) quality factor of MB-A*4 with various layers. (**c**) Filtration efficiency and pressure drop under various airflow rates. (**d**) Filtration efficiency and pressure drop during the loading test.

We also found that the filtration performances of our melt blown nonwovens varied with the areal density. According to Figure 10a, the filtration efficiencies increased with the increment of areal density, and samples with stepwise incremental gram weights of 10, 20, 30, 40, 50, and 60 g m^−2^ were 53.31%, 80.58%, 93.07%, 99.03%, 99.17%, and 99.36%, meanwhile the corresponding pressure drops were 20.58, 41.16, 62.72, 82.32, 101.92, and 125.44 Pa, respectively. These results indicated a synchronous increase of sample upon improving the areal density. It could be clearly seen that the filtration efficiencies presented a sharp rise, and then reached a plateau when the areal density exceeded 40 g m^−2^, while the pressure drop maintained at a low level and increased linearly with the increment of areal density. The QF values of materials fluctuated significantly, and the sample of 40 g m^−2^ shows the highest value of 0.056 Pa^−1^ (Figure 10b). By optimizing the processing parameters and adjusting the material structure, we obtained MB-A*4-0.4 sample with the best filtration performances. Therefore, a comprehensive evaluation on the filtration performances of MB-A*4-0.4 sample was further studied.

The air volume passed through the fiber material was another critical parameter for the overall filtration performances. Considering the complex application conditions of filters, the relationship between filtration performances and air flow rates (30-100 L min^−1^) was systematically measured. As showed in Figure 10c, with the increase of air flow rate, the filtration efficiency decreased gradually and finally reached 97.35% at the air flow of 100 L min^−1^. This result could be attributed to the reduced retention time of particles in the melt blown materials caused by higher air flux, which directly reduced the possibility for particles to collide on the fibers through Brownian diffusion. Moreover, we observed an almost linear correlation between pressure drop and air flow, which was consistent with Darcy’s law for viscous resistance. The slope of the linear fit of pressure drop versus air flow rate was only 0.88, which was much smaller than those of the previously reported fiber materials (2.03 for PA-6, 1.57 for N6-15 NFN) [27,28], indicating an excellent air permeability of our nonwovens in practical applications.

To investigate the dynamic filtration performances of sample, we also carried out the measurement of loading filtration. The mass median diameter of sodium chloride aerosol particles was 0.26 μm, and the concentration of aerosol particles was 20 mg m^−3^. In daily life, when PM_2.5_ pollution is in the most serious level, its concentration is only 0.5 mg m^−3^. This value is 40 times smaller than the concentration used in the testing condition, and thus indicating that the actual using time of our melt blown nonwovens can be extended to 5 h, which is acceptable for most applications. Figure 10d suggests that with the increase of loading time, the filtration efficiency of MB-A*4-0.4 decreased first and then increased, while the pressure drop kept increasing. The lowest point of filtration efficiency appeared at 4 min. At this time, the filtration efficiency was still greater than 95%, which meets the standard of N95 mask. This was due to the fact that at the beginning, the charge was shielded, resulting in the reduction in filtration efficiency. With more NaCl particles deposited on the surface of the material, a filter cake was formed and greatly increased the filtration efficiency [29,30].

## 4. Conclusions

In summary, multi-layered melt blown nonwovens with high filtration efficiency and ultralow pressure drop were successfully fabricated. Melt blown technology enabled the materials to form a 3D structure with fine fiber, small pore size but high porosity. Adding MgSt can change the crystal structure of PP to avoid charge escape and ensure the stability of filtration performance. The use of corona charging technology made different layers of nonwovens stick together and provided excellent filtration efficiency. The melt blown materials formed by four layers presented a significant filtration efficiency of 97.96%, a low pressure drop of 84.28 Pa, a high QF of 0.046 Pa^−1^ for paraffin oil aerosol particles. Meanwhile, a robust filtration efficiency of 99.03%, a low pressure drop of 82.32 Pa, an excellent QF of 0.056 Pa^−1^ for NaCl aerosol particles could be easily achieved (see Table S1 from Supplementary). It is usually more desirable for consumers and mask manufacturers to maintain high filtration efficiency and reduce pressure drop. Therefore, we anticipate that the multi-layered melt blown filtration material developed in this work would be applied in the field of protective masks.

## Figures and Tables

**Table 1:** Major processing parameters of melt blown.

Screw Zone 1 Temperature (°C)	Screw Zone 2 Temperature (°C)	Screw Zone 3 Temperature (°C)	Air Temperature (°C)	Distance (mm)	Screw Speed(r/min)	Air Pressure (MPa)
280	340	345	300	100	3	0.3

## Supplementary Materials

The following are available online at https://www.mdpi.com/2073-4360/13/4/485/s1, Table S1: Original date.
